# Phenotype and Genotype Study of Chinese *POMT2*-Related α-Dystroglycanopathy

**DOI:** 10.3389/fgene.2021.692479

**Published:** 2021-08-03

**Authors:** Xiao-Yu Chen, Dan-Yu Song, Li Jiang, Dan-Dan Tan, Yi-Dan Liu, Jie-Yu Liu, Xing-Zhi Chang, Guo-Gang Xing, Tatsushi Toda, Hui Xiong

**Affiliations:** ^1^Department of Pediatrics, Peking University First Hospital, Beijing, China; ^2^Department of Neurology, Children’s Hospital of Chongqing Medical University, Chongqing, China; ^3^Department of Neurobiology, School of Basic Medical Sciences, Peking University Health Science Center, Beijing, China; ^4^Department of Neurology, Graduate School of Medicine, The University of Tokyo, Tokyo, Japan

**Keywords:** dystroglycanopathy, mannosyltransferase, *POMT2*, genotype, phenotype

## Abstract

**Objective:**

Alpha-dystroglycanopathy (α-DGP) is a subtype of muscular dystrophy caused by defects in the posttranslational glycosylation of α-dystroglycan (α-DG). Our study aimed to summarize the clinical and genetic features of *POMT2*-related α-DGP in a cohort of patients in China.

**Methods:**

Pedigrees, clinical data, and laboratory tests of patients diagnosed with *POMT2*-related α-DGP were analyzed retrospectively. The pathogenicity of variants in *POMT2* were predicted by bioinformatics software. The variants with uncertain significance were verified by further analysis.

**Results:**

The 11 patients, comprising eight males and three females, were from nine non-consanguineous families. They exhibited different degrees of muscle weakness, ambulation, and intellectual impairment. Among them, three had a muscle-eye-brain disease (MEB)-like phenotype, five presented congenital muscular dystrophy with intellectual disability (CMD-ID), and three presented limb-girdle muscular dystrophy (LGMD). Overall, nine novel variants of *POMT2*, including two non-sense, one frameshift and six missense variants, were identified. The pathogenicity of two missense variants, c.1891G > C and c.874G > C, was uncertain based on bioinformatics software prediction. *In vitro* minigene analysis showed that c.1891G > C affects the splicing of *POMT2*. Immunofluorescence staining with the IIH6C4 antibody of muscle biopsy from the patient carrying the c.874G > C variant showed an apparent lack of expression.

**Conclusion:**

This study summarizes the clinical and genetic characteristics of a cohort of *POMT2*-related α-DGP patients in China for the first time, expanding the mutational spectrum of the disease. Further study of the pathogenicity of some missense variants based on enzyme activity detection is needed.

## Introduction

Alpha-dystroglycanopathy (α-DGP) is a subtype of congenital muscular dystrophy (CMD) with autosomal recessive inheritance. It is caused by defective O-mannosylglycosylation of α-dystroglycan (α-DG), which is required for binding to extracellular matrix ligands, such as laminins, neurexin, and perlecan. At least 18 pathogenic genes associated with α-DGP have been identified to date (Endo, 2015). *POMT2*, which encodes protein O-mannosyltransferase 2 (POMT2), is one of the pathogenic genes of α-DGP. It catalyzes the initial step in the biosynthesis of the α-DG O-mannosylation glycan chain, transferring mannose to serine or threonine residues (Willer et al., 2002). α-DGP shows high clinical and genetic heterogeneity. The most severe phenotype of *POMT2*-related α-DGP is Walker-Warburg syndrome [WWS, (OMIM 236670)], with an onset of intrauterine or after birth. WWS manifests as progressive muscle weakness and severe brain and eye abnormalities, such as lissencephaly, hydrocephalus, severe cerebellar involvement, complete or partial absence of the corpus callosum, congenital cataracts, glaucoma, and microphthalmia. Most patients cannot obtain ambulation ability and die before 1–3 years of age (Cormand et al., 2001; van Reeuwijk et al., 2005). The second severe phenotype is muscle-eye-brain disease [MEB, (OMIM 253280)], with milder brain structural abnormalities than WWS, including pachygyria, polymicrogyria, cerebellar, and brainstem dysplasia. A small number of MEB patients can acquire the ability to walk alone, although most patients have language and intellectual disability (Diesen et al., 2004). The intermediate phenotype is congenital muscular dystrophy with intellectual disability [CMD-ID, (OMIM 613156)]. Patients with this phenotype often have obvious intellectual and cognitive impairment, but their brain structure can be normal or only shows microcephaly and mild cerebral white matter changes (Godfrey et al., 2007). The mildest type of the disease is limb-girdle muscular dystrophy (LGMD) type 2N [LGMD2N, (OMIM 613158)], which presents a late onset and mild muscle weakness (Saredi et al., 2014; Østergaard et al., 2018). In this study, we summarized the results of clinical and mutational analyses of 11 *POMT2*-related α-DGP patients.

## Materials and Methods

### Patients and Clinical Data

This study [2015(916)] was approved by the Ethics Committee of the Peking University First Hospital. Written informed consent was obtained from the individuals and legal guardians for participation in the study as well as for the publication of any potentially identifiable images or data included in this article.

The inclusion criteria for α-DGP were described in our previous study (Song et al., 2020). In this study, patients with molecular genetic results indicating *POMT2* variants were enrolled, and patients diagnosed with other types of CMD or with other gene variants were excluded.

The patients’ clinical symptoms, physical examination results, and laboratory and molecular genetic tests were analyzed retrospectively.

### Molecular Genetic Testing

Genomic DNA was extracted from peripheral blood lymphocytes of the patients and their parents for whole exome sequencing (WES). Sequencing analysis was based on the reference sequence of *POMT2* (NM_013382.5), and the candidate variant was confirmed by Sanger sequencing in family members. We used some disease association databases, including the Human Gene Mutation Database (HGMD^1^), Leiden Open Variation Database (LOVD^2^), ClinVar database^3^, and 1,000 Genomes^4^ to identify pathogenic, clinically reported or novel mutations. If WES data revealed differences in the number of sequence reads between the patients and control samples, the change was considered to be a copy number variant (CNV), which was then confirmed by quantitative polymerase chain reaction (qPCR). The pathogenicity of novel or clinically unreported variants was predicted by using three kinds of bioinformatics software: MutationTaster^5^, PolyPhen2^6^, and Provean^7^.

### Muscle Biopsy and Immunofluorescence Staining

Open biopsy of the quadriceps or biceps brachii was performed, and the samples were fixed in isopentane precooled in liquid nitrogen. Routine histochemical staining was performed. Frozen sections (6 μm) from the muscle biopsy specimens were fixed with 4% paraformaldehyde at room temperature for 10 min. Non-specific binding was reduced by a 1-h incubation with 10% goat serum (Solarbio, China) in phosphate-buffered saline (PBS). The sections were then incubated at 4°C overnight with α-DG glycosylation antibody, IIH6C4 (Millipore, United States, 1:200), and β-DG antibody (Abcam, United Kingdom, 1:200), washed three times with PBS and then incubated with a goat anti-mouse fluorescent antibody (Life Technologies Corporation, United States, 1:500) at room temperature for 1 h. Immunofluorescence staining was observed by using an Olympus Fluoview FV10i (Olympus, Japan).

### Minigene Construction and Splicing Analysis

A hybrid minigene splicing assay *in vitro* was employed to evaluate the transcriptional effect of the variant on splicing. Wild-type and mutant minigene constructs comprising a genomic fragment spanning exon 17 to the exon 20 of *POMT2* were synthesized and cloned into the pcDNA 3.1 plasmid (Ge et al., 2019); the mutant minigene contains the variant c.1891G > C. Wild-type and mutant minigenes were transiently transfected into HEK-293 cells using Lipofectamine 3000 transfection reagent (Thermo Fisher Scientific, United States), and the cells were then incubated for 72 h before isolation of total RNA using TRIzol. cDNA was synthesized using 1 μg of RNA with a reverse transcription system kit (Promega, A3500, United States) and specific primers, including primers upstream in exon 17 (forward 5′-GGTCAATGACACAGATTTCCGA-3′) and downstream in exon 20 (reverse 5′-TAGGCAGTTCCCAGGAGCA-3′).

## Results

### Clinical Characteristics

A total of 11 *POMT2*-related α-DGP patients diagnosed by WES were enrolled. They come from non-consanguineous families, including eight males and three females. Among them, patients 4a and 5a were siblings, as were patients 7b and 8b. The patients were divided into three groups based on their clinical manifestations: MEB-like, CMD-ID, and LGMD2N. The clinical characteristics of these 11 patients were summarized in Table 1.

**TABLE 1:** Clinical characteristics of 11 *POMT2*-related alpha-dystroglycanopathy (α-DGP) patients.

Patient	1	2	3	4a	5a	6	7b	8b	9	10	11
Gender	Male	Male	Male	Female	Male	Female	Male	Male	Male	Male	Female
Last follow-up age	12 years	5 years	11 years	11 years	6 years	21 years	11 years	8 years	9 years	4 years	4 years
Onset age	3–4 months	After birth	4 years	4 months	8 months	18 months	3–4 months	3–4 months	3 years 5 months	3–4 months	5 months
Phenotype	CMD-ID	CMD-ID	LGMD	MEB-like	MEB-like	LGMD	CMD-ID	CMD-ID	LGMD	MEB-like	CMD-ID
Initial symptom	Developmental delay	Developmental delay	Abnormal gait	Developmental delay	Developmental delay	Walking unstably	Developmental delay	Developmental delay	Limbs weakness	Developmental delay	HyperCKemia
Maximal mobility	Run and jump (worse)	Sit without support	Run and jump (slightly poor)	Sit without support	Sit without support	Run (unstable)	Walk independently	Walk independently (unstable)	Run and jump	Sit with support	Walk independently
Joint contractures	No	Yes	No	Yes	Yes	No	Yes	Yes	No	Yes	No
Language development	Normal	Single word	Normal	Single word	Single word	Dysarthria	Words and short sentences	Words and short sentences	Poor expression	Unconscious syllables	Short sentences
Intelligence disability	Yes	Yes	Yes	Yes	Yes	Yes	Yes	Yes	Yes	Yes	Yes
Head circumference/percentile	<P_3_	<P_25_	<P_3_	<P_3_	<P_3_	<P_3_	<P_3_	NA	<P_3_	<P_3_	<P_3_
CK (IU/L)	2,480–7,774	2,000–4,683	3,200–5,193	3,609–5,200	Not detected	2,760–8,000	6,808–9,080	4,956	1,362–2,204	3,656–4,602	3,152–4,347
Cardiovascular involvement	No	No	No	No	Not detected	No	No	No	No	No	No
Ocular involvement	No	Esotropia	No	No	No	No	No	No	No	Esotropia	No
Electromyogram	Myogenic change	Myogenic change	Not done	Myogenic change	Not done	Normal	Not done	Normal	Non-specific change	Not done	Myogenic change
Muscle biopsy	Muscular dystrophic change	Not done	Not done	Muscular dystrophic change	Not done	Muscular dystrophic change	Muscular dystrophic change	Not done	Slightly pathogenic change	Not done	Not done
Brain MRI	Slightly enlarged subarachnoid space, slightly deeper sulcus	Slightly enlarged bilateral ventricles	Normal	Cerebellar and brainstem dysplasia	Not done	Slightly enlarged posterior horn of the lateral ventricle	Extensive signal abnormalities in the white matter of the brain	Abnormal white matter signal in frontal and parietal lobe and periventricular	Abnormal white matter signal in posterior body of lateral ventricle	Polymicrogyria, cerebellar and brainstem dysplasia, cerebellar cysts, enlarged bilateral ventricles	Normal
Genetic test	c.287A > G p.(Tyr96Cys) c.551C > T p.(Thr184Met)	c.1237C > T p.(Arg413X) c.604T > G p.(Phe202Val)	c.479A > G p.(Tyr160Cys) c.287A > G p.(Tyr96Cys)	c.1521C > A p.(Tyr507X) c.227T > G p.(Leu76Trp)	c.1521C > A p.(Tyr507X) c.227T > G p.(Leu76Trp)	c.1891G > C p.(Gly631Arg) c.295C > T p.(Arg99Cys)	c.874G > C p.(Ala292Pro), homozygote	c.874G > C p.(Ala292Pro) homozygote	c.2176G > A p.(Gly726Arg) c.1261C > T p.(Arg421Trp)	c.1769_1772dupATCT p.(Leu592Ser fs*189) c.1769A > G p.(Tyr590Cys)	c.227T > G p.(Leu76Trp) c.1274G > A p.(Ser425Asn)
											

**FIGURE 1:**
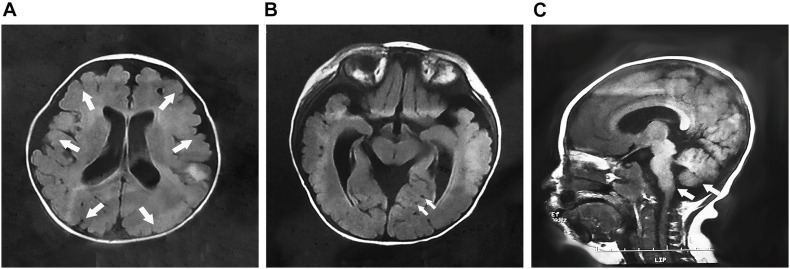
The brain MRI of patient 10 at 7 months of age. **(A)** Cross-sectional imaging showed polymicrogyria in the frontal, temporal, parietal, and occipital regions (arrows); **(B)** multiple cysts were identified in the cerebellum (arrows); and **(C)** sagittal imaging showed the cerebellum and brainstem dysplasia (arrows).

The MEB-like group includes patients 4a, 5a, and 10. Their age of onset was between 3 and 8 months old, manifesting as psychomotor developmental delay, microcephaly, and joint contractures. Their maximum mobile ability was only supported or unsupported sitting. Patients 4a and 5a were only able to speak simple words; patient 10 could pronounce two syllables unconsciously. In addition, the patients were only able to understand simple instructions. All of them had no abnormal history of birth. Physical examination revealed obvious myopathy appearances, small head circumferences, and prominently decreased muscle strength in the proximal limbs. In patient 4a, multiple joint contractures including interphalangeal, knee, and ankle joints and severe scoliosis occurred from 4 years old. Patient 5a presented with pseudohypertrophy of the forearm and gastrocnemius and knee joint contracture at the age of 5 years. Patient 10 showed a high palate arch and knee joint contracture at the age of 2 years. However, eye involvement in three children was not obvious, with only patient 10 exhibiting esotropia. Their serum creatine kinase (CK) levels were moderately elevated and fluctuated between 3,609 and 5,200 IU/L. Brain MRI showed cerebellar and brainstem dysplasia in patients 4a and 10 and obvious polymicrogyria in patient 10 (Figure 1). As the clinical and MRI data of these three patients were similar to MEB, but milder than it, we considered their phenotype to be MEB-like.

The CMD-ID group includes patients 1, 2, 7b, 8b, and 11. Their age of onset was between shortly after birth and 5 months of age. The symptoms initially manifested as motor developmental delays with different degrees of severity. The maximum motor capacity of patient 1 was running and jumping but worse than that of normal children of the same age; patient 2 could only sit without support. Patients 7b, 8b, and 11 were able to walk alone, albeit unstably, and patient 7b had lost his ambulant capacity at the age of 10 years due to severe knee and ankle joint contractures. All five patients exhibited intellectual disability. Patients 1 and 7b had difficulty learning at school and poor reading ability. Patients 2, 8b, and 11 were only able to understand simple instructions. Although patient 1’s language development was generally normal, the remaining patients could only speak single words or short sentences. Physical examination revealed muscle weakness, which was dominant in the proximal lower limbs. Patient 7b exhibited pseudohypertrophy in the gastrocnemius muscle with a positive Gowers’ sign and obvious knee and ankle joint contractures at the age of 10 years. Patient 2 displayed slight esotropia; the remaining patients showed no ocular involvement. Their CK levels ranged from 2,000 to 9,080 IU/L. Brain MRI of these patients showed no structural abnormalities, though patients 1 and 2 revealed an enlarged subarachnoid space or ventricle, and patients 7b and 8b showed abnormal white matter signals.

The LGMD2N group includes patients 3, 6, and 9. Their onset age ranged from 1 to 4 years old, with initial manifestations of abnormal or unstable gait and limb muscle weakness that were relatively milder than those of the patients above. They were susceptible to fatigue, although their daily activities were not affected, and they had poorer running and jumping abilities than normal individuals of the same age. All of them had difficulties learning at school and poor reading skills. The language development of the three patients was generally normal, but patient 6 had mild dysarthria, and patient 9 had poor language expression. Physical examination at the age of last follow-up revealed small head circumferences and slightly decreased muscle strength, which was proximally dominant, with no joint contractures. The patellar tendon reflex of patient 6 was weakened and that of patients 3 and 9 was not drawn out. They had no ocular involvement currently. CK levels ranged from 1,362 to 8,000 IU/L in these patients. Brain MRI indicated normal cerebral structure, though the posterior horns of the lateral ventricle of patient 6 were enlarged slightly, and patient 9 had abnormal white matter signals. Considering the late onset age, mild clinical symptoms, unlimited daily activities and lack of brain structural abnormalities of the three patients, we categorized them into the LGMD group. Notably, we found that all patients had learning difficulties through follow-up, as it was difficult for them to understand the contents of courses, and they had poor mathematical calculation and memory skills.

### Molecular Genetic Analysis

A total of 15 different *POMT2* variants, including 9 novel variants and 6 known variants were identified in these 11 patients. No pathogenic CNVs were found. The genetic data for the 11 patients were summarized in Table 1. We performed pathogenicity prediction for the 11 unreported variants using bioinformatics software. Most of the variants are missense changes in highly conservative regions. Nine of these 11 variants are predicted to be deleterious or likely pathogenic, but the pathogenicity of 2 missense variants, c.1891G > C and c.874G > C, are uncertain (Table 2). Base G at position c.1891 is the last base in exon 18 of the *POMT2* gene, and a nucleotide change at this position would possibly affect the splicing of the transcript. The position c.874G is not particularly conservative, and this change may be benign based on the prediction of software. In order to confirm the pathogenicity of these two variants, we have conducted further research.

**TABLE 2:** Pathogenicity prediction results of clinically unreported *POMT2* variants.

Variants/software	Mutationtaster	PolyPhen 2	Provean
c.287A > G	Pathogenic	Probably damaging (score: 1)	Deleterious (score: −8.127)
p.(Tyr96Cys)			
c.1237C > T	Pathogenic	/	/
p.(Arg413X)			
c.479A > G	Pathogenic	Probably damaging (score: 1)	Deleterious (score: −6.068)
p.(Tyr160Cys)			
c.1521C > A	Pathogenic	/	/
p.(Tyr507X)			
c.227T > G	Pathogenic	Probably damaging (score: 0.999)	Deleterious (score: −4.154)
p.(Leu76Trp)			
c.1891G > C	Pathogenic	Benign (score: 0.001)	Neutral (score: 0.382)
p.(Gly631Arg)			
c.295C > T	Pathogenic	Probably damaging (score: 1)	Deleterious (score: −6.274)
p.(Arg99Cys)			
c.874G > C	Polymorphism	Possibly damaging (score: 0.564)	Neutral (score: −2.35)
p.(Ala292Pro)			
c.1769_1772dupATCT	Pathogenic	/	/
p.(Leu592Ser fs*189)			
c.1769A > G	Pathogenic	Probably damaging (score: 1)	Deleterious (score: −8.066)
p.(Tyr590Cys)			
c.1274G > A	Pathogenic	Probably damaging (score: 0.996)	Deleterious (score: −2.8)

### Muscle Pathology and Immunofluorescence Staining

**FIGURE 2:**
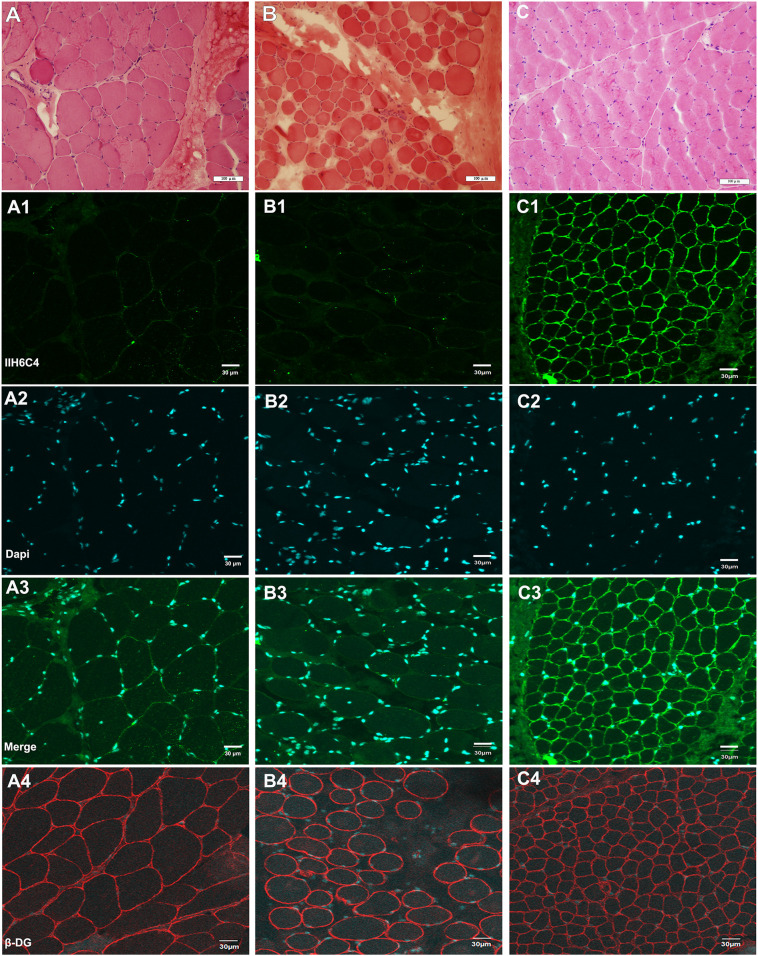
Hematoxylin–eosin (HE) staining and immunofluorescence staining of IIH6C4 antibody of patients 6 and 7b **(A,B)** HE staining of patients 6, and 7b showed slightly increased variation of the muscle fiber diameter, and necrosis; **(C)** HE staining of the healthy control; **(A1,B1,C1)** Immunofluorescence staining of IIH6C4 antibody from patients 6, 7b, and the normal control, respectively; **(A2,B2,C2)** the staining of Dapi for cell nucleus of patients 6, 7b, and the normal control, respectively; **(A3,B3,C3)** the staining of merge, and the staining of patients 6 and 7b showed the expression of glycosylated α-DG in the cell membranes was much weaker than in the control; and **(A4,B4,C4)** the staining of β-DG antibody for patients 6, 7b, and the normal control, respectively.

Hematoxylin–eosin (HE) staining of the left biceps brachii biopsy of patient 6 showed muscular dystrophic changes with slightly increased variation in muscle fiber diameter, necrosis, and regeneration of muscle fibers accompanied by inflammatory cell infiltration (Figure 2A). In addition, obviously proliferated lipids and connective tissue in the partial perimysium, increased variation in muscle fiber diameter, and some necrotic muscle fibers were detected in the muscle biopsy of the right quadriceps of patient 7b (Figure 2B). Based on immunofluorescence staining with an IIH6C4 antibody, the expression of glycosylated α-DG in the muscle cell membrane from patients was significantly defective compared to that in the control (Figures 2A1–C3). The staining of β-DG antibody was normal in both patients and the healthy control (Figures 2A4–C4).

### Minigene Assay Analysis

**FIGURE 3:**
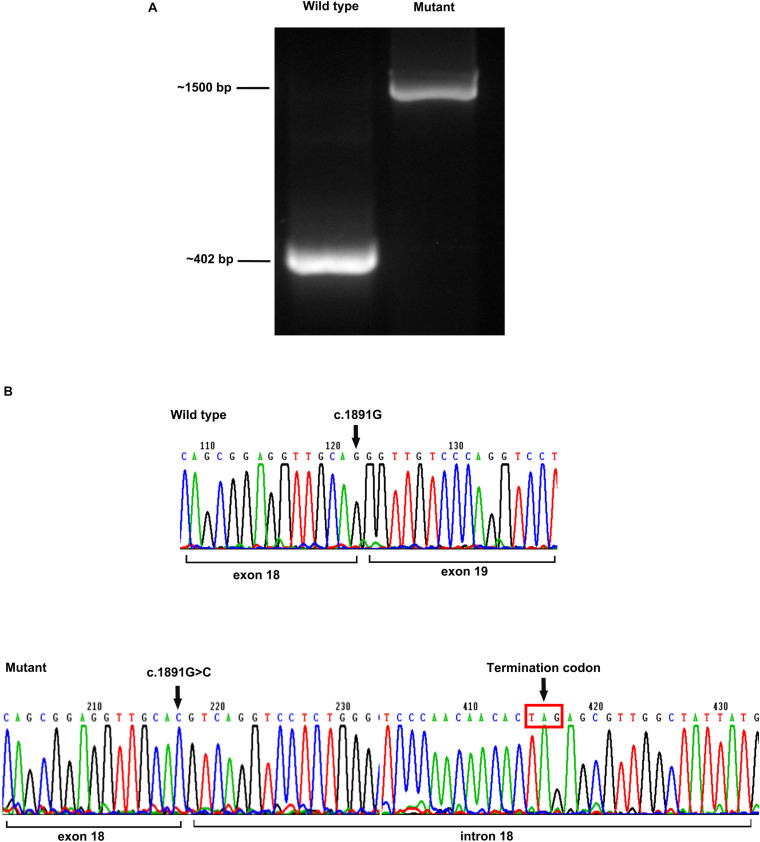
Minigene assay analysis. **(A)** The size of an electropherogram of PCR product of the wildtype was around 402 bp, and the size of mutant was around 1,500 bp; and **(B)** sequencing of the cDNA revealed the intron 18 was spliced into the mutant, resulting to the premature appearance of termination codon.

A minigene of *POMT2* carrying the c.1891G > C variant was constructed to verify the transcriptional consequences of the splice site change. An electropherogram of PCR products revealed the predicted size 402-bp band in the wild-type control, whereas a larger 1,500-bp band was observed in the mutant (Figure 3A). Sequencing of the PCR product showed that the intron between exons 18 and 19 was spliced retention, which suggested that the variant c.1891G > C affects splicing. Furthermore, we found a termination codon when analyzing the sequence of the retention intron 18, which probably resulted in the premature termination of the protein translation (Figure 3B).

## Discussion

As *POMT2* catalyzes the first step in O-mannosylation of α-DG, it is believed that *POMT2* mutations would cause severe phenotypes, and the first case of *POMT2*-related α-DGP presented the most severe phenotype of WWS, as expected (van Reeuwijk et al., 2005). With an increasing number of genetically diagnosed cases reported, a number of milder phenotypes of *POMT2*-related α-DGPs have been identified, such as MEB/FCMD-like (Godfrey et al., 2007), CMD-ID (Yanagisawa et al., 2007), and LGMD2N (Biancheri et al., 2007; Murakami et al., 2009; Saredi et al., 2014; Østergaard et al., 2018). However, due to the pleiotropic effects of the genes involved, there is no definite correlation between genotype and phenotype, and the same mutation site in different individuals may cause variable phenotypes. Although patients with *POMT2*-related α-DGP present variable phenotypes, they still have common features, including slowly progressive muscle weakness, predominantly in the proximal muscle group, elevated CK levels, and psychomotor retardation.

The development of next-generation sequencing technology enhances the diagnostic rate. Here, we summarize the characteristics of a *POMT2*-related α-DGP cohort in China. Their age of onset ranged from after birth to childhood. The phenotype included LGMD, CMD-ID, and MEB-like, but the most severe phenotype WWS was not seen in this cohort. All the patients developed slowly progressive muscle weakness and intellectual disability with varying degrees and no severe ocular involvement at the last follow-up. All of them had microcephaly and none of them has developed epilepsy to date, although some only exhibited white matter signal abnormalities on brain MRI and no brain structural abnormalities. Some studies have suggested that the results of brain MRI are not necessarily related to intellectual and cognitive impairment (Palmieri et al., 2011). In general, intellectual disability in α-DGP possibly results from defective α-DG glycosylation in neurons, inhibiting the long-term potentiation of the hippocampus and ultimately leading to learning and memory dysfunction (Satz et al., 2010).

**FIGURE 4:**
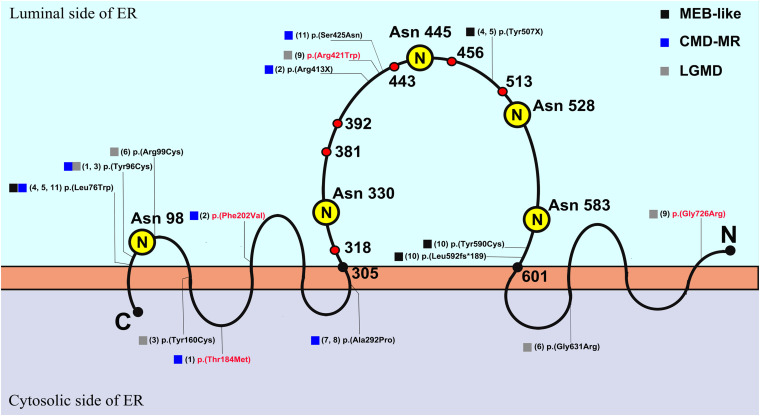
The schematic diagram of *POMT2* (Manya et al., 2010) and the distribution of variants in this cohort. The capital letter *N* in yellow circle represented N-glycosylation sites, which are located on the amino acids of Asn 98, Asn 330, Asn 445, Asn 528, and Asn 583. The amino acids 305–601 represented the large hydrophilic loop, and it contains three presumed MIR domains, amino acid 318–381, 392–443, and 456–513, which are the important areas that affect enzymatic activity. The number in brackets represented the serial number of patients, and the variants in red font had been clinically reported; ER, endoplasmic reticulum; C, C terminal; N, N terminal.

*POMT2* is a member of the protein O-mannosyltransferase family, and it is relatively conserved in different species from yeast to humans. The protein, containing nine transmembrane regions, catalyzes O-mannosylation, which is essential for skeletal muscle function and neuronal migration (Willer et al., 2002). Formation of a heterodimer with its homologous analog, POMT1, is necessary for *POMT2* to exert mannosyltransferase function (Manya et al., 2004). When synthesis of the glycosylated glycan chain is completed, α-DG can act as a receptor for the binding of many ligands in the extracellular matrix, and this receptor function plays a key role in stabilizing the cytoskeleton, completing material transportation, and signal transduction. The enzyme activity of *POMT2* depends on five N-glycosylation sites, namely, Asn98, Asn330, Asn445, Asn528, and Asn583, which are all located on the luminal side of the endoplasmic reticulum. The last four sites are located in a large hydrophilic loop, containing amino acids 305–601, which are key areas that affect *POMT2* enzymatic activity (Manya et al., 2010). There are three presumed homologous domains in this hydrophilic loop, called the mannosyltransferase-3-phosphate inositol receptor-ryanodine receptor (M-IP3R-RyR, MIR) domain, each consisting of amino acids 318–381, 392–443, and 456–513 (Figure 4); these domains may also be important for the catalytic function of *POMT2* (Ponting, 2000).

As p.(Arg413X) in patient 2, p.(Tyr507X) in patients 4a and 5a, p.(Arg421Trp) in patient 9, p.(Leu592Ser fs^∗^189), and p.(Tyr590Cys) in patient 10 in this study are located in the large hydrophilic loop or MIR domain of *POMT2*, they are likely to be pathogenic based on their position. Nonetheless, it is difficult to predict the effect of the remaining variants on protein function according to their sites. c.1891G > C/p.(Gly631Arg) in patient 6 is not located in an area critical for enzymatic activity, and the software analysis also predicted that it is possibly non-pathogenic. However, this variant is located at the end of exon 18, so we suspected it may affect splicing of *POMT2*. We then constructed a minigene carrying the variant c.1891G > C to verify its pathogenicity. We confirmed that this missense change of the last base in exon 18 leads to a splicing change and premature termination of the protein translation. Homozygous c.874G > C in patients 7b and 8b was predicted to be polymorphic or benign, but immunofluorescence staining from patient 7b’s skeletal muscle biopsy confirmed hypoglycosylation of α-DG, suggesting the pathogenicity of this variant.

Four compound heterozygous variants, p.(Thr184Met), p.(Phe202Val), p.(Gly726Arg), and p.(Arg421Trp), in our cohort have been reported previously. The phenotype of patient 1 with variants of p.(Thr184Met) and p.(Tyr96Cys) manifested as CMD-ID, though in a previous report, the phenotype of an individual also harboring heterozygous variants of p.(Thr184Met) and p.(Trp748Ser) was LGMD (Godfrey et al., 2007). Patient 2 carrying p.(Phe202Val) manifested as CMD-ID, consistent with a previous report about a homozygote of p.(Phe202Val) (Murakami et al., 2009). The phenotype of homozygous p.(Gly726Arg) in a previous report was WWS (Bouchet et al., 2007), and the heterozygous variant p.(Arg421Trp) caused variable phenotype (Punetha et al., 2016; Johnson et al., 2018). Patient 9, with a compound heterozygous variant of p.(Gly726Arg) and p.(Arg421Trp), presented the phenotype of LGMD. We marked the variant sites of our cohort on the *POMT2* protein schematic illustrated in Figure 4. In general, if the variant is located on the luminal side of the endoplasmic reticulum, especially on the large hydrophilic loop related to the enzyme activity of *POMT2*, it usually causes a more severe phenotype. If the variant is located on the cytoplasmic side, it may cause a milder phenotype due to the slight impact on the enzyme catalytic activity. However, this is not absolute, as clinical severity depends on how the mutation site affects protein function.

Our study broadens the mutational spectrum of *POMT2*-related α-DGP, but there is no clear correlation between genotype and phenotype. The main mutational types in this study were missense variants, and most of them were compound heterozygotes. It is difficult to determine the pathogenicity of a missense variant, even though it is located in the catalytic domain of the protein, as there may be some residual protein function. Hence, further study is needed to detect whether the enzymatic activity of *POMT2* is affected by the missense variants described here.

## Data Availability Statement

The datasets for this article are not publicly available due to concerns regarding participant/patient anonymity. Requests to access the datasets should be directed to the corresponding author.

## Ethics Statement

The studies involving human participants were reviewed and approved by the Ethics Committee of the Peking University First Hospital. Written informed consent to participate in this study was provided by the participants’ legal guardian/next of kin. Written informed consent was obtained from the individual(s), and minor(s)’ legal guardian/next of kin, for the publication of any potentially identifiable images or data included in this article.

## Author Contributions

X-YC: conceptualization, methodology, validation, investigation, and writing – original draft. D-YS: resources and formal analysis. LJ and Y-DL: resources. D-DT: formal analysis. J-YL and X-ZC: investigation. G-GX and TT: technique support. HX: writing, review and editing, supervision, project administration, and funding acquisition. All authors contributed to the article and approved the submitted version.

## Conflict of Interest

The authors declare that the research was conducted in the absence of any commercial or financial relationships that could be construed as a potential conflict of interest.

## Publisher’s Note

All claims expressed in this article are solely those of the authors and do not necessarily represent those of their affiliated organizations, or those of the publisher, the editors and the reviewers. Any product that may be evaluated in this article, or claim that may be made by its manufacturer, is not guaranteed or endorsed by the publisher.

## Footnotes

1
http://www.hgmd.cf.ac.uk/ac/index.php

2
http://www.dmd.nl/

3
https://www.ncbi.nlm.nih.gov/clinvar/

4
http://www.1000genomes.org/

5
http://www.mutationtaster.org/

6
http://genetics.bwh.harvard.edu/pph2/

7
http://provean.jcvi.org/
